# On the Education of Dentists

**Published:** 1845-12

**Authors:** 


### ARTICLE VIII.

### *On the Education of Dentists.*

No one can legally practice as a surgeon who has not gone through a certain curriculum of education, produced certificates, at least of having attended lectures, and passed an examination, such as it is, at the College of Surgeons. Dentism, on the contrary, has no legal restrictions, any one who can afford to purchase a brass plate, and a few second-hand instruments, may write dentist after his name, and victimize the public *ad libitum* —nay, by constantly advertising, may keep up a fresh supply of victims, although he be as ignorant as a horse—his whole qualifications consisting in a little tact, and an unlimited supply of brass; for though dentism frequently involves mistakes and injury, they are seldom accompanied by danger, and most people, but more especially females, prefer bearing any inconvenience arising from incompetence or dishonesty to exposing to their dearest friends how much they are indebted to art for their beauties.

From this and some other causes there is no profession so laden with charlatanism and quackery as that of dental surgery, and if its respectable professors do not adopt some means to stop the constant influx of ignorance and incompetence it will soon be completely overrun, and the preponderance of the bad bring it into perfect contempt.

We know of one man who prepares dental practitioners with more than railroad speed—who has the unblushing effrontery to promise to fit them for the profession in one month—to teach them the whole art and science of dentism, both surgical and mechanical, in 26 days; and this, not requiring their constant attendance, but two hours twice or three times a week. After this very elaborate preparation, which is in some few instances extended to the enormous period of three months, he gives his pupils certificates of competence; surely he must mean incompetence, and ushers them into the world as perfect dentists—certificates of competence, the competence of 48 hours study.

We ask any man of common sense and common honesty what such certificates can be worth, and yet the public consider them as infallible, and employ their possessors without further question or inquiry. Can any man, however talented, even though he be an M.R.C.S. into the bargain, obtain a knowledge of the first principles of the rudiments of the art in so ridiculously inadequate a time, much less obtain the power of performing mechanical operations that require constant attention and great assiduity to acquire at all; and yet this creature offers to teach any one, whatever may have been his previous employment, whether he has been a carpenter or a milkman, the whole art in one little month, "Ere those shoes were old," &c. and turn them out full-blown dentists after one course of each of his lectures, and this for the sake of a few paltry pounds. Verily we should like to see some of the handicraft of these 48 hour dentists.

Can any profession hope to be respectable that is afflicted with such an incubus, that not only has the power but employs it to the utmost of inflicting such gross injustice on its members, and such unprincipled injury on the public, and yet he is not a solitary instance of grasping infamy, there are others, who, though they pursue their plans less ostentatiously, cause equal mischief.

Are the respectable practitioners so blind that they do not see what is known to half the world—are they content so long as they amass fortunes themselves, to let the public be gulled and victimized as the villainy and the opportunities of these unprincipled men may dictate? Are they so reckless of their own honor that they can wink at such things, and consent to be recognized of the same class with these fellows? Are they content that their art should be considered a nonentity, and *their* pretence to knowledge a cheat upon the public?

They cannot be so absurdly foolish as to suppose there is no remedy for such a state of things. Why, then, are they so culpably inert as not to embrace it? Every accident that occurs, every imposition that is practised may fairly be imputed to them if they wilfully or negligently let things remain as they are.

But it may be asked, how are these practices to be prevented? Certainly not, by trusting to the desperate chance of any improvement in the morals or the practice of these men—a profession commenced in deception will be carried on with dishonesty. Nor by thrusting their hands into their pockets and jingling their fees to a de Frieschutz accompaniment; to do any thing they must be up and stirring. Although they at *present* have no legal powers, nothing but unanimity and determination are wanting to obtain them, and is the art they practice of such slight importance, or are they so disreputable or uninfluential as a body that they either fear or do not think it worth while to make the attempt?

We are not advocates for monopoly, but for fair and wholesome restrictions that will at once protect the interests of the profession and the public, who at present cannot judge of the competence of a dental practitioner until they have employed him, and may attribute the failures of ignorance to imperfections in the art itself, rather than to the incompetence of men whom they believe accredited practitioners.

Few people have either time or inclination to inquire how a man has obtained his education, they see that he is allowed to practice openly, and unacquainted with the peculiarities of the profession, take it for granted that he is competent to do so, though at present they have no security for this competence. The being a member of the College of Surgeons is none, for, strange to say, a man may be at the same time a very excellent surgeon, but an execrable dentist.

It is the duty, therefore, of the practitioners of the dental art, to take such steps as will at once protect themselves and the public, and the straightforward, and indeed only way to do this, is to form a society or association. Once established, and there could be no earthly reason, why the government should refuse to arm it with the necessary powers.

We allow that the effects of a dental association would be prospective, rather than present, and as no acts of parliament are retrospective, that many would necessarily be admitted to its advantages who would have no just claim to enjoy them; but it would, to a certain extent, ensure the respectability of the profession, and the competence of the next generation of practitioners, and by so doing, give a higher tone to the profession. We remember that the effects of the Apothecaries Act of 1815, were felt, (however imperfectly it has since been carried out,) in the increased respectability of the profession, long before it could have produced any actual change in the members. A dental association would act still more quickly, by preventing the wholesale manufacture of practitioners.

An imperative condition of an association of this kind, must be, that (excepting those in practice) no one should be allowed to practice as a dentist, who had not gone through a proper and efficient course of study, and passed such an examination, both theoretical and practical, as will ensure, as far as such precautions can, his competence to perform his duties.

The curriculum of education should be fixed on a liberal scale—it should not be such as would ensure mere capability, but should embrace anatomy, physiology, pathology and surgery, as far as these sciences are in the most remote degree connected with dental surgery in its most extended sense. The principles of mechanics should be studied, not as applied to the dental art *alone*, but in their broad and fundamental principles, and to this should be added an acquaintance with geometry and mathematics; and while chemistry, as affording a wide field for energy and research, should not be omitted, a knowledge of the French and German languages, would be found of the greatest advantage.

Modelling, and other processes involved in the practice of the mechanical part of the art, and which would form a necessary part of the instruction, would of course come under the department of the private teacher. To superior minds, many other studies, such as comparative anatomy, &c. would necessarily suggest themselves, and which, though not essential to the practice of dental surgery, mark the man of education and science.

This course of education might be carried out by the establishment of normal schools on the one hand, and lectureships and a *dental*, similar to the opthalmic institution, on the other—a dispensary devoted to the teeth, and *open to all*, where the student might acquire a knowledge and experience in the different diseases and imperfections of the teeth and gums—the imperfections of the palate, and every branch of dental surgery; tracing their symptoms, watching the different modes of treatment, and learning to remedy, mechanically, those imperfections in which curative means are of no avail.

But the nice mechanical arrangements—the mode of fastening and finishing mechanical contrivances, in short *the handicraft* of the profession, can only be acquired by perseverance, attention, and *practice*, at the work-bench of a professor; and consequently a probation, varying as to time, from three to five years, either as an apprentice or a pupil, is essential to the formation of a perfect dentist.

There is no time to spare, every day is adding fresh members to the profession, who can bring nothing but disgrace in their train—the go-ahead system is rapidly increasing, and it may soon be too late to interfere with any chance of a favorable result.—*London Forceps.*

